# Normalized cumulative gain as an alternative evaluation measure for genomic selection models

**DOI:** 10.1186/s12711-025-01022-9

**Published:** 2025-12-16

**Authors:** Felix Heinrich, Thomas M. Lange, Faisal Ramzan, Mehmet Gültas, Armin O. Schmitt

**Affiliations:** 1 Breeding Informatics Group, Department of Animal Sciences, Georg-August University, Göttingen, Margarethe von Wrangell-Weg 7, 37075 Göttingen, Germany; 2 Institute of Animal and Dairy Sciences, University of Agriculture Faisalabad, Jail Road, 38000 Faisalabad, Pakistan; 3 Faculty of Agriculture, South Westphalia University of Applied Sciences, 59494 Soest, Germany; 4 Center for Integrated Breeding Research (CiBreed), Albrecht-Thaer-Weg 3, 37075 Göttingen, Germany

## Abstract

****Background**:**

Genomic selection relies on a variety of statistical and machine learning methods to predict phenotypes from genomic data. Since no single method consistently outperforms others across datasets, evaluating and comparing model performance is essential. However, standard evaluation metrics such as Pearson’s correlation coefficient and mean squared error treat genomic prediction as a regression problem, assessing overall fit rather than the effectiveness of selecting top-performing individuals for breeding. This disconnect can lead to suboptimal model selection in practice.

****Results**:**

To address this, we present the normalized cumulative gain (NCG) as an alternative evaluation measure that directly measures the phenotypic gain achieved from the individuals selected by the model. We applied this measure on four animal and plant datasets to compare nine commonly used methods for genomic prediction.

****Conclusions**:**

NCG offers an intuitive and interpretable measure of selection efficiency, focusing solely on the individuals that would actually be chosen. We further demonstrate that calculating the performance under all possible selection thresholds provides more information than a single or few arbitrary thresholds. This more granular analysis shows that the performance of the methods may differ under varying selection intensities and can provide guidance for the choice of selection intensity. Our approach is implemented in R and is available at https://github.com/FelixHeinrich/GS_Comparison_with_NCG.

## Background

With new advances in the field of sequencing massively decreasing the cost and time necessary for genotyping an individual [1, 2], genomic prediction (GP) has become an increasingly important tool in animal and plant breeding [3–5]. Using phenotypes that are predicted based on genetic markers covering the whole genome, individuals can be selected for further breeding, a breeding strategy that is referred to as genomic selection (GS) [6]. This approach has been successfully applied across a wide range of species to accelerate their genetic progress [7–10]. An abundance of different algorithms have been proposed for genomic prediction. These algorithms include among others, linear regression-based methods such as Best Linear Unbiased Prediction (BLUP), various Bayesian approaches (e.g. BayesA, BayesB, BayesC, Bayesian Lasso, and Bayesian Ridge Regression), and machine learning models such as Gradient Boosting, eXtreme Gradient Boosting, and Random Forest [11–13]. To date, no single method has consistently outperformed the others across all datasets; model performance is highly dependent on the specific characteristics of the dataset under study [12, 14]. It is therefore essential to compare multiple models on a given dataset to determine which performs best.

The most commonly used metric for model evaluation in GP is Pearson’s correlation coefficient (*r*) between predicted and observed phenotypes [13, 15, 16]. Other widely used measures include the coefficient of determination ($R^2$) and mean squared error (MSE) [12, 14, 17]. These measures are standard in regression analysis and focus on assessing the overall predictive accuracy of the model [18, 19]. Combined with the selection intensity, *r* determines the expected genetic gain according to the Breeder’s Equation and thus reflects the theoretical success of genomic selection. However, such global measures overlook the core objective of GS which is selecting the top *k* individuals for breeding, where *k* may be a fixed number or a proportion of the population. In this context, predicted phenotypes are only an intermediate output. The success of GS depends primarily on whether individuals with the highest true phenotypes are among those which were selected. Measures like MSE or *r* may be heavily influenced by the accurate ranking of low-performing individuals, which has little to no bearing on GS outcomes. While *r* can be interpreted as selection accuracy, using it to predict the rate of genetic gain requires the assumption of normally distributed breeding values. This assumption may not hold, as many agronomic traits – such as the number of weeds or the percentage of infested leaf area – exhibit non-normal phenotypic distributions [20, 21]. In addition, true breeding values, although theoretically measurable, must in practice be inferred from phenotypic data under specific model assumptions [22]. Moreover, perfect ordering of individuals is possible even without a perfect correlation ($r = 1$), further reducing the utility of correlation-based evaluation in some cases [23].

The limitations of these commonly used evaluation metrics in GS have been previously highlighted by Blondel et al. [23], who proposed reframing GS as a ranking problem. They introduced a metric from the field of information retrieval – Normalized Discounted Cumulative Gain (NDCG) – to assess model performance [24]. Unlike measures such as *r* or MSE, which treat all individuals equally, NDCG emphasizes only the top *k* individuals intended for selection. Without delving into the full mathematical formulation, the measure compares the phenotypes of individuals selected based on predicted values with those that would be selected if true phenotypes were known. However, a disadvantage of NDCG is its use of a discounting function, which penalizes incorrect ordering among the selected individuals. While this makes sense in information retrieval contexts (e.g., search engines where top-ranked results should be most relevant), it is less applicable to GS, where only the inclusion or exclusion of an individual in the selected set matters—not their exact ranking within it. A related measure, Relative Efficiency (RE), introduced by Ornella et al. [25], measures the ratio between the expected genetic gain from individuals selected using predicted values and the gain from those selected based on true phenotypes. Despite some methodological differences, both NDCG and RE focus exclusively on the selected individuals, aligning more closely with the objectives of GS. However, in previous studies, these metrics were often reported only for a few specific values of *k*, leaving the model’s performance across a broader range of selection thresholds unexplored.

In this study, we present the Normalized Cumulative Gain (NCG) as an evaluation measure for the effectiveness of GS. Like NDCG and RE, it focuses solely on the selected individuals, but it avoids the interpretability issues associated with discounting. NCG offers a more intuitive understanding of how well a model performs in identifying the most valuable candidates for selection. To provide a more comprehensive evaluation, we propose assessing model performance across all possible values of *k*, rather than limiting the analysis to a few arbitrary thresholds. This more nuanced perspective makes it possible to identify which models perform best within specific selection ranges. It may also help inform decisions about selection thresholds for breeding programs by taking into account not only economic factors but also the possible gain achieved through GS. We apply this approach to four publicly available datasets using nine commonly used genomic prediction algorithms.

## Methods

To describe the calculation of the NCG measure, we consider a test dataset comprising the true phenotypes $Y$ and the predicted phenotypes $\hat{Y}$ for *n* individuals, where $Y_{i}$ and $\hat{Y}_i$ denote the true and predicted phenotypes for individual *i*, respectively.

Using notation similar to [26], let $y(i, Y)$ denote the *i*-th phenotypic observation sorted in descending order according to the true phenotypes $Y$, such that $y(1,Y)$ is the highest phenotype, $y(2, Y)$ the second highest, and $y(k, Y)$ the *k*-th highest. Let $y(i, \hat{Y})$ denote the *i*-th phenotypic observation sorted in descending order according to the predicted phenotypes $\hat{Y}$. Specifically, $y(k, \hat{Y})$ is the true phenotype of the individual ranked *k*-th by the predictions $\hat{Y}$ of the model. The NCG for selecting the top *k* individuals is then defined as

1 $$\begin{aligned} NCG@k(Y,\hat{Y}) = \frac{\sum _{i=1}^{k}{y(i, \hat{Y})}}{\sum _{i=1}^{k}{y(i, Y)}} \end{aligned}$$

In comparison, the NDCG by Blondel et al. [23] is calculated as

2 $$\begin{aligned} NDCG@k(Y,\hat{Y}) = \frac{\sum _{i=1}^{k}{g(y(i, \hat{Y}))d(i)}}{\sum _{i=1}^{k}{g(y(i, Y))d(i)}} \end{aligned}$$

where $g(y)$ is a monotonically increasing gain function (e.g., $g(y) = y$ for linear gains) and $d(i)$ is a monotonically decreasing discount function (e.g., $d(i) = \frac{1}{\log _{2}(i+1)}$). A related measure is the Mean-NDCG@*K*, defined as the mean of the NDCG values from $k = 1$ to *K*. The RE by Ornella et al. [25] is defined as

3 $$\begin{aligned} RE@k(Y,\hat{Y}) = \frac{\frac{\sum _{i=1}^{k}{y(i, \hat{Y})}}{k}-\frac{\sum _{i=1}^{n}{Y_{i}}}{n}}{\frac{\sum _{i=1}^{k}{y(i, Y)}}{k}-\frac{\sum _{i=1}^{n}{Y_{i}}}{n}} \end{aligned}$$

At their core, all three measures share a similar structure. Each compares the sum of the true phenotypes of the top *k* individuals selected based on predictions to that of the true top *k* individuals. This normalization ensures that the maximum achievable value is 1, indicating perfect selection.

We compared the performance of nine commonly used methods for genomic prediction: Ridge Regression BLUP (BLUP), Bayes A (BA), Bayes B (BB), Bayes C (BC), Bayesian Lasso (BL), Bayesian Ridge Regression (BRR), Gradient Boosting Machine (GBM), eXtreme Gradient Boosting (XGBoost) and Random Forest (RF). BLUP and the Bayesian methods are linear models, implemented in the R packages rrBLUP 4.6.3 [27] and BGLR 1.1.3 [28], respectively. The primary differences among these linear methods lie in their approaches to variable selection and shrinkage of coefficients, as detailed by de los Campos et al. [29]. The three machine learning methods – GBM, XGBoost and RF – are all ensemble-based algorithms that combine the outputs of multiple weaker learners to enhance predictive performance. RF trains multiple independent regression trees and aggregates their predictions via averaging [30]. In contrast, GBM [31] and XGBoost [32] build models sequentially, with each successive learner trained to improve the predictions of the previous one [33]. RF was implemented in the R package ranger 0.17 [34], while GBM and XGBoost were implemented in the gbm 2.2.2 [35] and xgboost 1.7.7.1 [36] packages, respectively. All methods were applied using their default parameter settings.

We evaluated the performance of GS using the NCG measure on four publicly available genotype-phenotype datasets from both animal and plant species. To ensure an unbiased comparison across genomic prediction methods, all SNPs with missing genotype values were removed prior to analysis. as the methods differ in their ability to handle missing data.

Chicken: The first dataset consists of egg weight records for 1063 Rhode Island Red chickens (*Gallus domesticus*) measured at 36 weeks of age [37]. Genotyping was performed using the Affymetrix Axiom^®^ 600 K Chicken Genotyping Array, resulting in 294,705 SNPs for analysis.

Goats: The second dataset, provided by Guan et al. [38], includes 210-day milk yield records for 822 Murciano-Granadina goats (*Capra hircus*). Genotyping was conducted using the Goat SNP50 BeadChip [39], yielding 33,133 SNPs.

Rice: This dataset includes 937 rice lines (*Oryza sativa* L.) from Uruguay [40]. Since the lines were tested across multiple field trials between 1997 and 2020, we used the average grain yield per line as the phenotype. Genotypes for each line were obtained via genotyping-by-sequencing, resulting in 61,260 SNPs.

Wheat: The final dataset pertains to the grain yield of 498 recombinant inbred lines of wheat (*Triticum aestivum* L.) [41], planted during the 2017-2018 growing season. Genotyping was carried out using the Axiom^®^ 35k Wheat Breeder’s genotyping array [42]. Following the approach of Lange et al. [43], SNPs with a minor allele frequency of $\le$1% and those with more than 10% missing values were excluded. Remaining missing values were imputed using LinkImpute 1.1.4 [44].

Following previous studies [12, 23, 45], we used cross-validation to evaluate the ability of the models to predict unobserved phenotypes. The individuals were randomly partitioned into five folds, with each fold serving once as the test set (20%) while the remaining four folds (80%) were used for training. To ensure a fair comparison, all models were trained and tested using the same data splits. This fivefold cross-validation procedure was repeated ten times, with the individuals randomly reshuffled before each repetition. In total, each model was evaluated across 50 train-test splits. For each run, performance was assessed using both the NCG and Pearson’s correlation coefficient (*r*), and the results were averaged across all runs to obtain the final performance estimates. As the *r* values were not normally distributed, we used the Kruskal–Wallis rank sum test followed by Dunn’s post hoc test to identify statistically significant differences in median *r* values among the models, using a significance threshold of $\alpha = 0.05$. The R package multcompView 0.1-9 [46, 47] was used to assign group labels to the models based on whether their performances differed significantly.

## Results

**Fig. 1:**
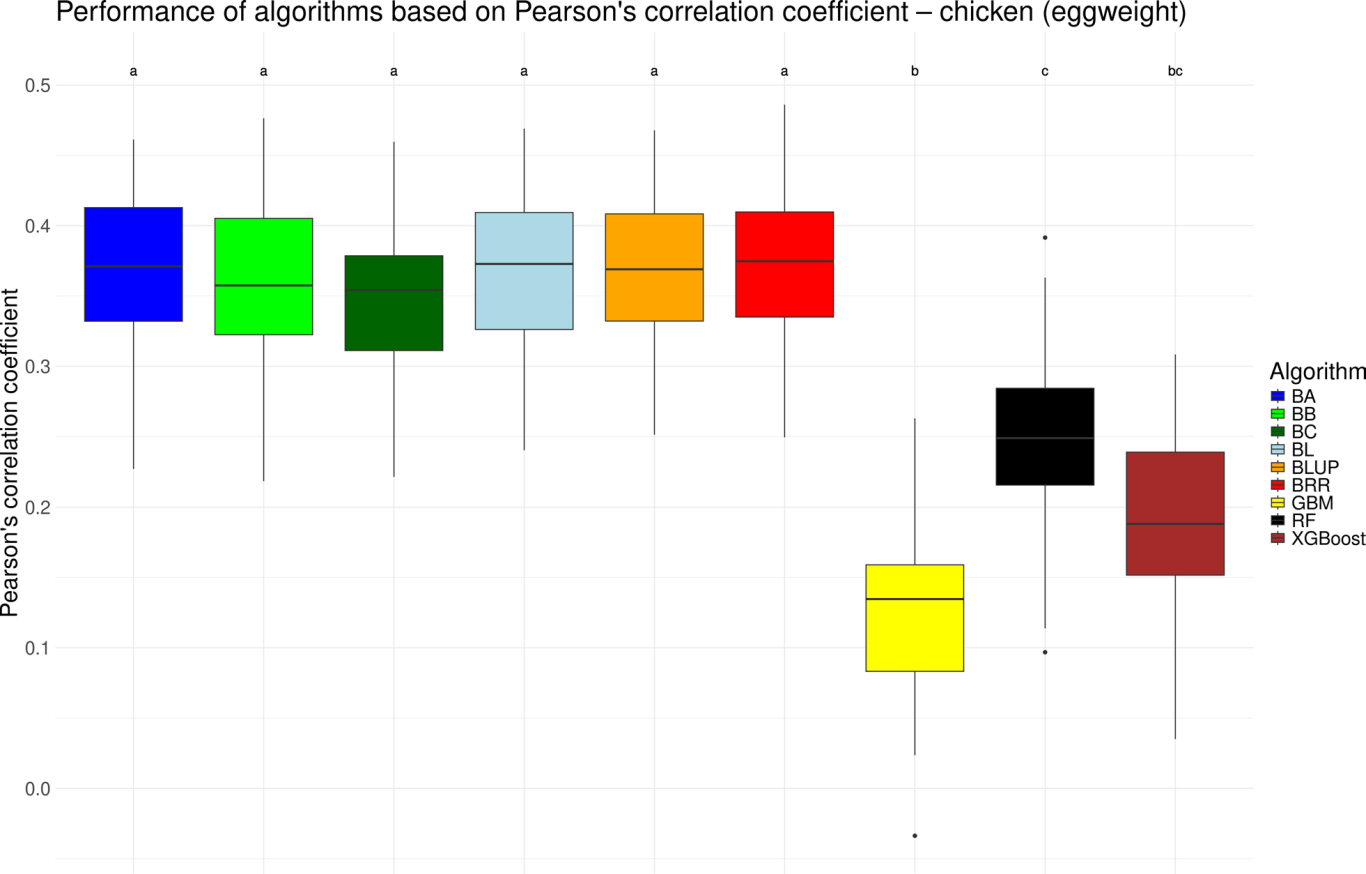
Performance of different genomic prediction algorithms on the chicken dataset, measured using Pearson’s correlation coefficient. Letters above the boxes indicate statistically significant differences between methods, with methods sharing the same letter not being significantly different ($\alpha = 0.05$)

**Fig. 2:**
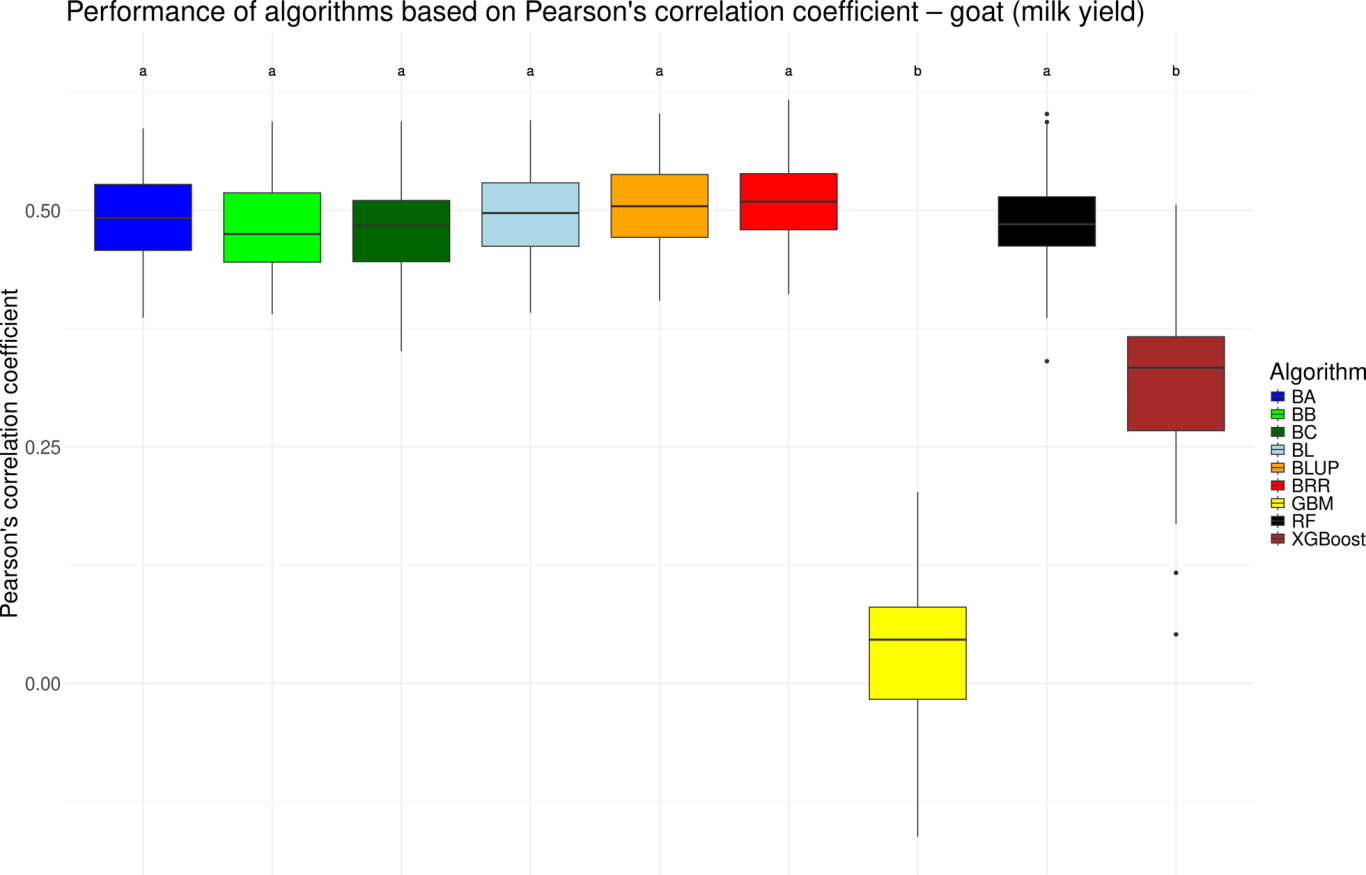
Performance of different genomic prediction algorithms on the goat dataset, measured using Pearson’s correlation coefficient. Letters above the boxes indicate statistically significant differences between methods, with methods sharing the same letter not being significantly different ($\alpha = 0.05$)

**Fig. 3:**
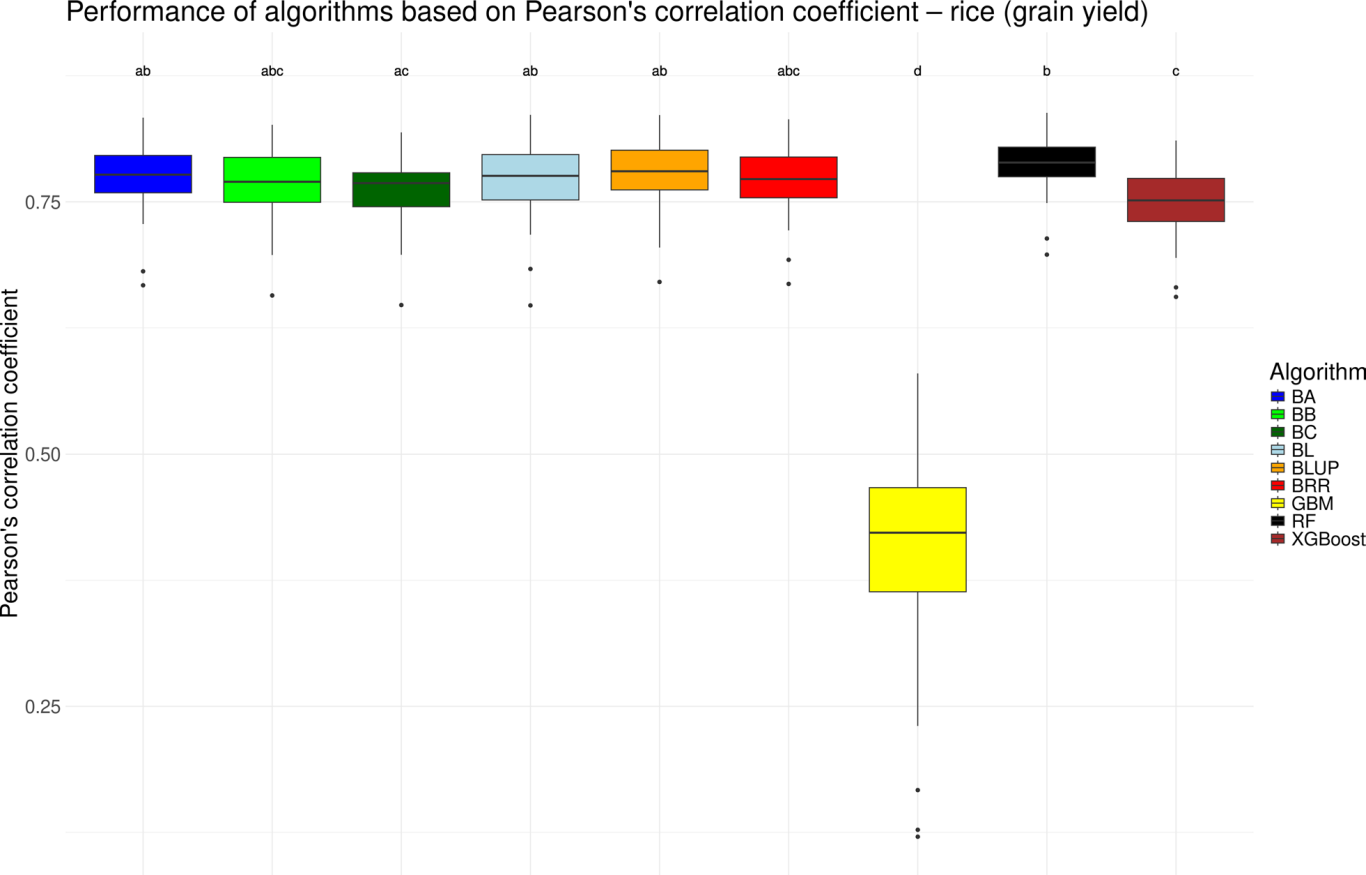
Performance of different genomic prediction algorithms on the rice dataset, measured using Pearson’s correlation coefficient. Letters above the boxes indicate statistically significant differences between methods, with methods sharing the same letter not being significantly different ($\alpha = 0.05$)

**Fig. 4:**
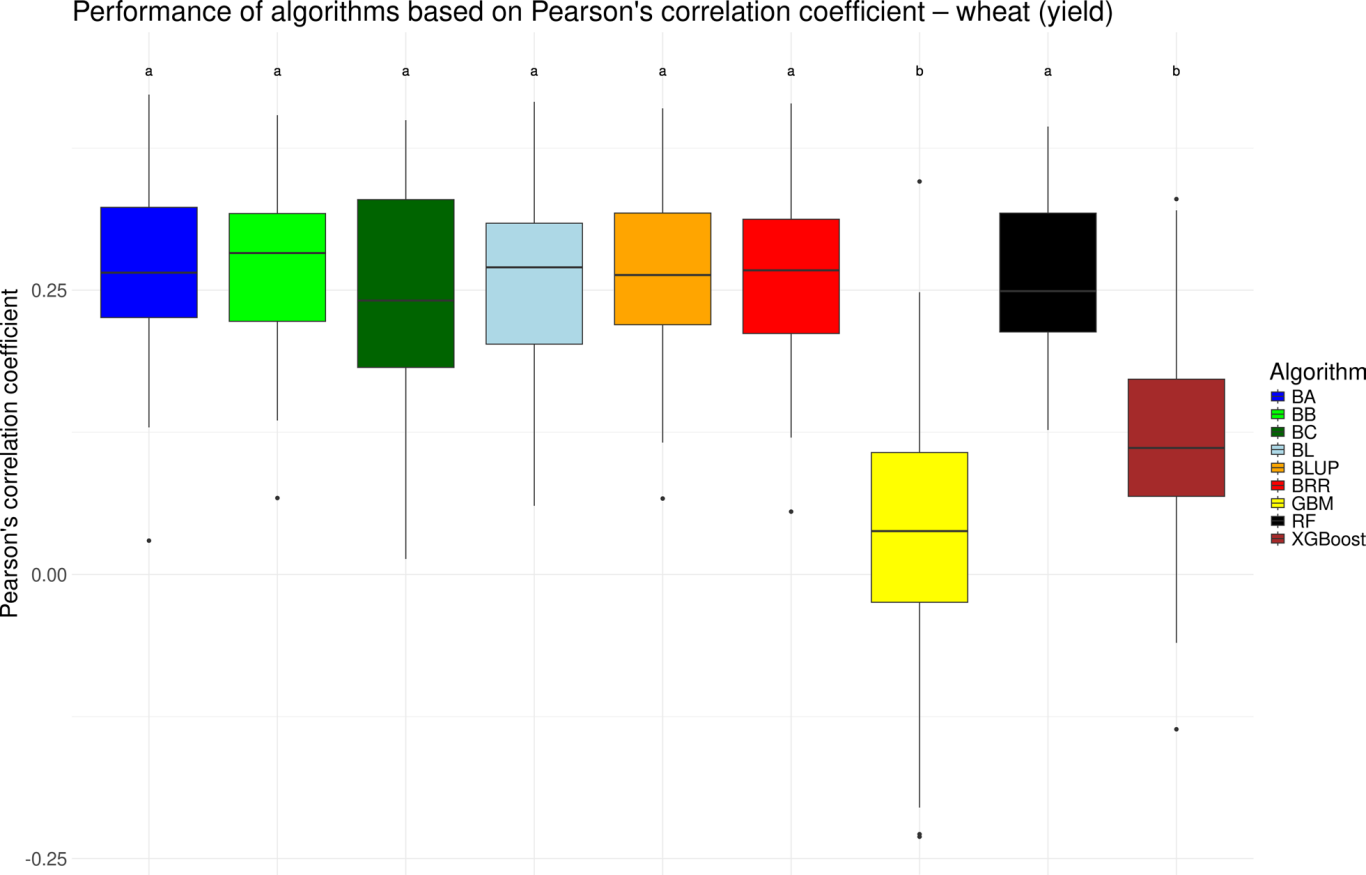
Performance of different genomic prediction algorithms on the wheat dataset, measured using Pearson’s correlation coefficient. Letters above the boxes indicate statistically significant differences between methods, with methods sharing the same letter not being significantly different ($\alpha = 0.05$)

We visualized the performance of various genomic prediction algorithms in the form of boxplots for the traditional measure of Pearson’s correlation coefficient *r* in Figs. 1, 2, 3, and 4 for the chicken, goat, rice and wheat datasets, respectively. As noted previously, the performance of genomic prediction models often depends on the specific dataset being analyzed [12, 14]. However, despite variation in correlation levels across the datasets, the relative performance of the algorithms was highly consistent. Across all four datasets, the seven linear models yielded statistically similar results. Among the non-linear machine learning methods (GBM, RF, and XGBoost) consistent trends emerged: GBM generally showed the lowest performance, RF achieved results comparable to the linear models, and XGBoost typically performed in between. These differences were partially statistically significant. Notable exceptions were observed in two datasets. In the chicken dataset, RF performed significantly worse than the linear models (see Fig. 1), while in the rice dataset, XGBoost matched the performance of the linear models but was still significantly outperformed by RF (see Fig. 3). Overall, based on Pearson’s correlation coefficient (*r*), no single algorithm significantly outperformed all others. These results suggest that any of the tested linear models, as well as RF (with the exception of the chicken dataset), would be appropriate for genomic prediction.

**Fig. 5:**
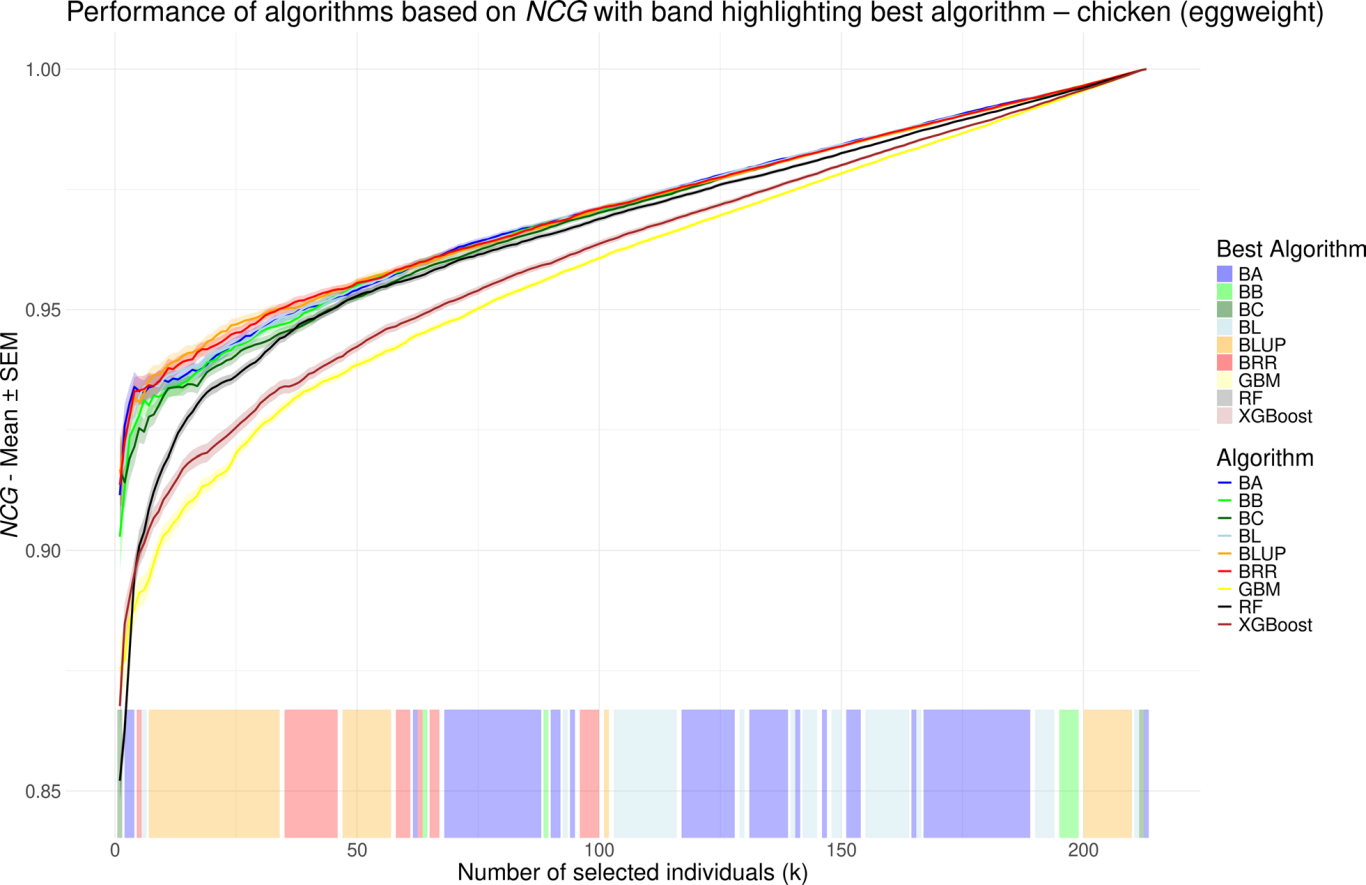
Performance of different genomic prediction algorithms depending on the number of selected individuals (*k*) on the chicken dataset, measured using normalized cumulative gain. Shaded ribbons indicate the standard error of the mean. The color of the shaded area at the bottom indicates the method with the highest NCG value for the corresponding *k* values

**Fig. 6:**
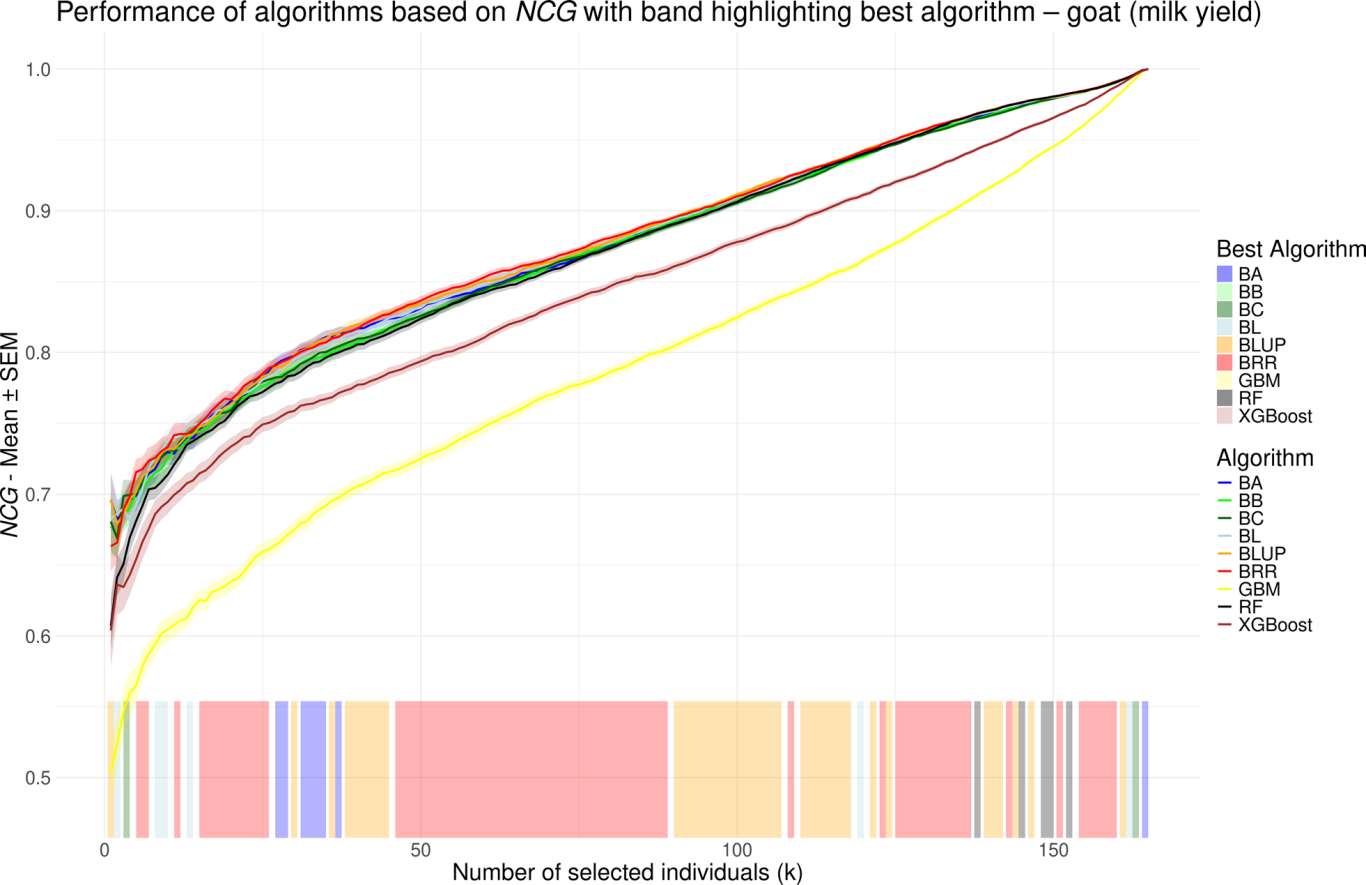
Performance of different genomic prediction algorithms depending on the number of selected individuals (*k*) on the goat dataset, measured using normalized cumulative gain. Shaded ribbons indicate the standard error of the mean. The color of the shaded area at the bottom indicates the method with the highest NCG value for the corresponding *k* values

**Fig. 7:**
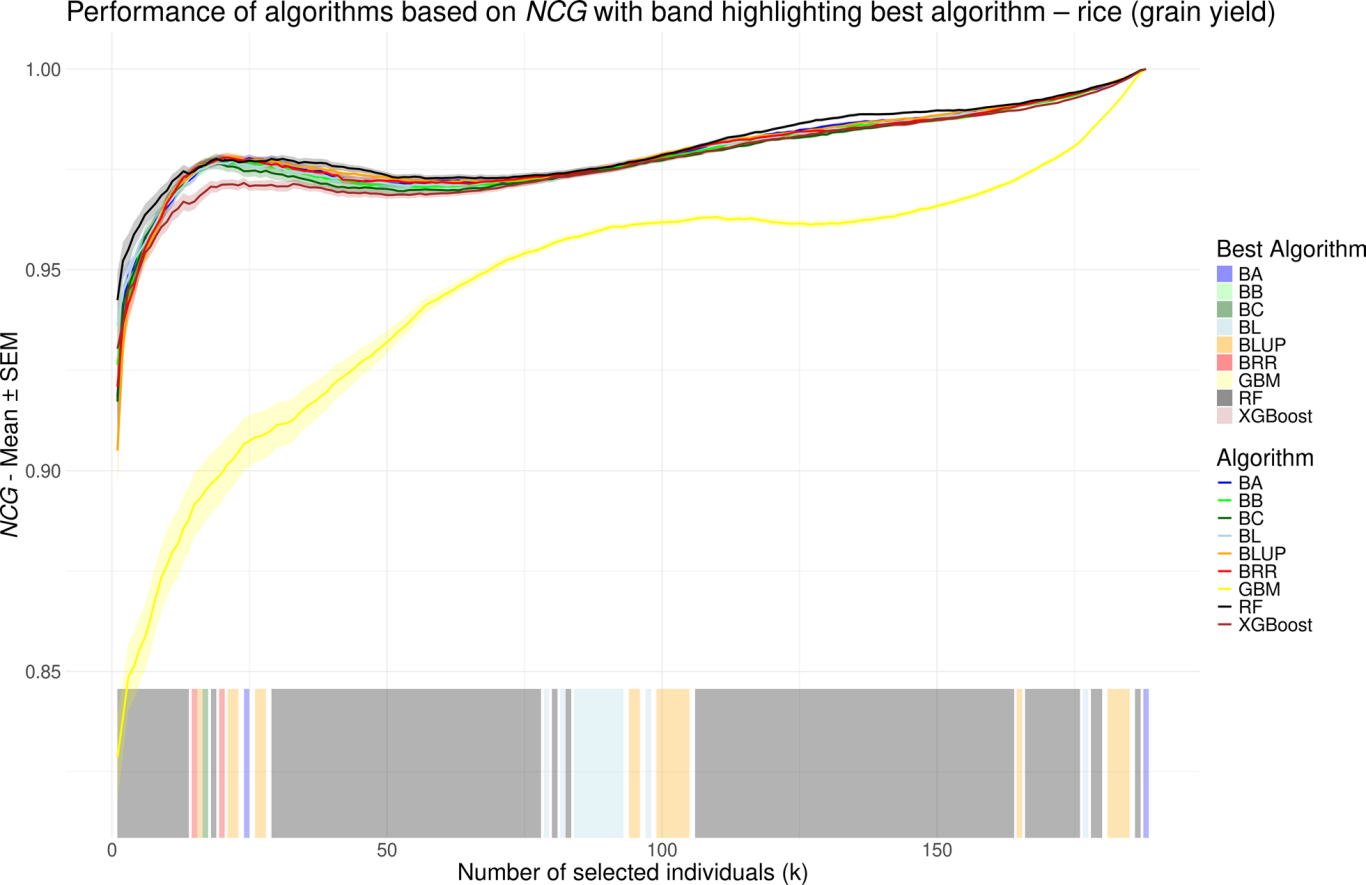
Performance of different genomic prediction algorithms depending on the number of selected individuals (*k*) on the rice dataset, measured using normalized cumulative gain. Shaded ribbons indicate the standard error of the mean. The color of the shaded area at the bottom indicates the method with the highest NCG value for the corresponding *k* values

**Fig. 8:**
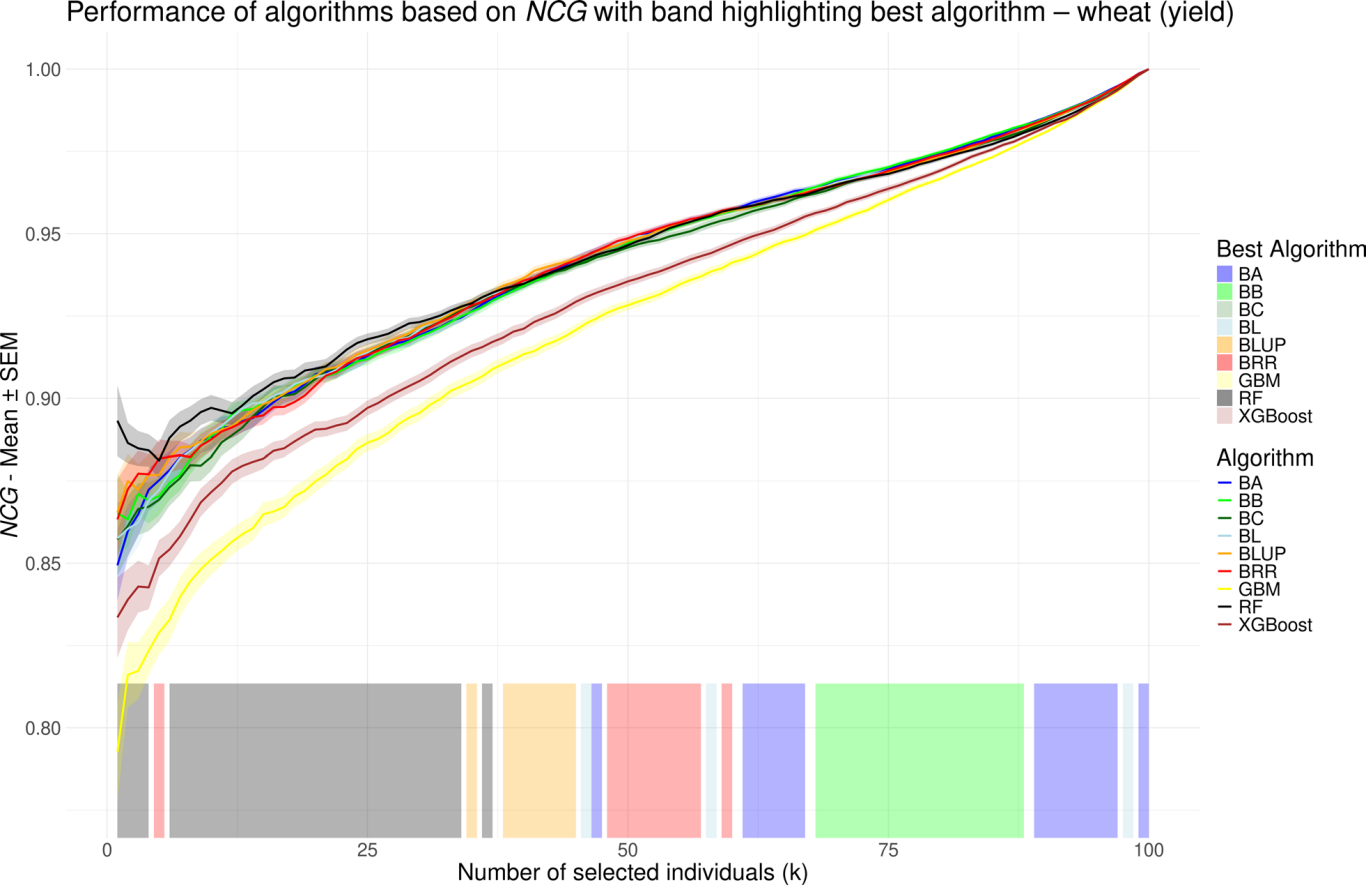
Performance of different genomic prediction algorithms depending on the number of selected individuals (*k*) on the wheat dataset, measured using normalized cumulative gain. Shaded ribbons indicate the standard error of the mean. The color of the shaded area at the bottom indicates the method with the highest NCG value for the corresponding *k* values

While *r* provides a single summary value for model performance, normalized cumulative gain (NCG) allows for performance evaluation at each possible selection size (*k*), i.e., the number of individuals to be selected. In contrast to previous studies [48–55] that used the NDCG measure by Blondel et al. [23] – most of which only reported performance for a limited number of selected *k* values – we aimed to compare model performance comprehensively across all possible *k* values. To this end, we visualized NCG values as line plots, similar to the approach used by Ma et al. [56] and Yu et al. [26]. NCG values, averaged over repeated cross validation runs, are shown in Figs. 5, 6, 7, and 8 for the chicken, goat, rice and wheat datasets, respectively. To facilitate visual interpretation of the best-performing model at each *k*, we added a shaded band along the bottom of each plot, color-coded to indicate which model achieved the highest NCG value at each selection size. The interpretation of NCG values is intuitive and straightforward. For instance, in the rice dataset, an NCG value of 0.977 at $k=25$ for the random forest model indicates that, on average, the sum of phenotypes for the 25 individuals selected by the model corresponds to 97.7% of the sum of phenotypes for the true top 25 individuals (see Fig. 7). When considering overall model performance, NCG provides results that are largely consistent with those obtained using *r*. The linear models and RF demonstrate the best and largely comparable performance, followed by XGBoost, which in some cases achieves NCG values similar to those from the linear models (e.g. Fig. 7). GBM, on the other hand, consistently performs the worst, although it achieves slightly higher NCG values than RF and XGBoost for $k < 5$ in the chicken dataset (see Fig. 5). The range of NCG values varies strongly across the four datasets. For example, GBM starts with an NCG value of 0.504 in the goat dataset, whereas initial NCG values in the other datasets range from 0.793 to 0.875. In the rice dataset (see Fig. 7), RF performs best for most *k* values, as indicated by the color-coded band at the bottom of the plot. In contrast, in the wheat dataset (see Fig. 8), RF outperforms the other models only for *k* in (1, 34), after which the best-performing model alternates between the linear models BLUP, BRR, BA and BB, which obtain highly similar values. Conversely, for the chicken and goat datasets (see Figs. 5 and 6, respectively), there is no large *k* interval where RF outperforms the other methods; instead, the linear models consistently reach the highest NCG values. However, the order and relative performance of models in these two datasets are different, with no shared pattern regarding which model performs best on a certain *k* interval.

## Discussion

While the overall performances based on NCG are similar to those obtained using *r*, the key advantage of NCG lies in its ability to evaluate model performance across the full range of selection sizes (*k*), offering a more granular perspective under varying levels of selection strength. For example, in the rice dataset, we observe specific *k* intervals – such as (1, 13), (31, 48) and (120, 150) in Fig. 7 – where the performance gap between the best and second-best models becomes more pronounced. The same can be seen for the interval (1, 13) in Fig. 8. In these intervals, selecting the top-performing model can yield a noticeable advantage, whereas in other ranges, the performance differences are minimal. Conversely, in Fig. 7, XGBoost performs noticeably worse than the linear methods for *k* values between approximately 10 and 35, while achieving comparable performance outside this range. The shaded band at the bottoms of Figs. 5, 6, 7, and 8, which color-codes the best-performing method at each *k*, highlights that the same model often maintains top performance across consecutive ranges of *k*. This includes both intervals with meaningful performance advantages and those where the margin is negligible (e.g., *k* in (37, 57) in Fig. 8). Therefore, careful inspection of the NCG curves of the respective models is necessary to assess whether the best performing model actually provides tangible advantages. In particular, the previously mentioned *k* intervals that show such advantages of a certain model are not transferable across the species or traits and instead are specific to the analyzed dataset. One could for example argue that there is no such *k* interval in Fig. 6 at all. This is in line with previous studies that found the overall performance of the models to be highly dataset-dependent [12, 14].

This approach contrasts with the previous studies by Ma et al. [56] and Yu et al. [26], which visualized the Mean-NDCG@*K* across *k* values ranging from 1% to 100%. While this allows for performance comparison, the resulting values are less interpretable for two reasons: (1) NDCG includes a discounting factor that is unnecessary in the context of GS, and (2) Mean-NDCG@*K* at $K=i$ depends on all NDCG@*k* values for $k \le i$, making it unsuitable as a standalone performance metric for selecting the top *i* individuals.

By definition, the NCG value for all models converges to 1 when *k* equals the population size—that is, when no selection is applied and all individuals are included. However, prior to this point, NCG values are not necessarily monotonically increasing. In fact, the addition of a single individual can sometimes result in a noticeable drop in performance. This behavior is most apparent in Fig. 8, where the RF model achieves an average NCG of 0.893 at $k = 1$, which decreases to 0.886 at $k = 2$. In other words, the model is slightly more effective at identifying the single best individual than it is at identifying the best two. Such fluctuations in performance are most pronounced at small values of *k* for two reasons. First, the selection targets the extreme tail of the phenotype distribution, which, under the properties of a normal distribution, contains individuals whose phenotype values are far above the mean; ranking errors in this region can therefore lead to large absolute losses of potential gain. Second, when *k* is small, the number of values that are summed up is limited, such that the contribution of each newly selected individual constitutes a large relative fraction of the total cumulative gain. Conversely, as *k* increases, these effects diminish, leading to a smoother and generally increasing curve. Consequently, the differences in performance between the GS models are also largest at small *k* values.

Nevertheless, NCG values do not always increase monotonically. For instance, in Fig. 7, all models except GBM exhibit a minor plateau in NCG values around $k = 25$, followed by a slight decline up to $k = 50$, before resuming a more typical upward trend. This pattern, which occurs only for the rice dataset, may support a more informed decision for selecting an appropriate value of *k*, as choosing to select more individuals along the decline may yield limited additional benefit despite increased resource expenditure. NCG provides an intuitive measure of the percentage difference between the cumulative gain achieved by selection based on model predictions and the theoretical optimum obtained by selection based on the true values. During the decline, this gap widens as individuals with increasingly larger discrepancies between predicted and true phenotypic values are added to the selection set. With respect to the performance differences across all *k* values between methods, particularly between GBM and the linear models, we observe that once the curves become smoother, the difference in NCG values does not follow a uniform pattern across the datasets. In Figs. 5 and 8, the difference decreases gradually, albeit at a slow rate. By contrast, in Fig. 6, the decline accelerates for the last 40 individuals. In Fig. 7, however, the difference reaches a local minimum at around $k=80$ before increasing again. This further emphasizes the value of calculating NCG across the entire range of *k*, as it reveals nuanced behaviors that would otherwise remain hidden.

It should be pointed out that the NCG measure is inherently designed for phenotypes where higher values are desirable, such as yield – as used in our datasets – or traits like protein content, where individuals with the highest phenotypic values should be selected. However, not all traits in animal and plant breeding follow this pattern. For many economically important phenotypes, including somatic cell count in milk or residual feed intake, lower values are more desirable [57, 58]. To appropriately apply NCG in these cases, a transformation of the phenotypic values is required so that higher values correspond to more favorable outcomes. One straightforward approach is to subtract each individual’s phenotype from the maximum observed phenotypic value, effectively inverting the scale while preserving relative differences.

It is also important to note that some studies have proposed binary classification metrics such as accuracy and Cohen’s kappa coefficient for evaluating genomic selection models [17, 25]. However, these metrics do not account for the actual phenotypic values of selected individuals and therefore fail to reflect the true impact of selecting individuals with suboptimal phenotypes due to inaccurate predictions. For this reason, we excluded them from our analysis.

We applied the NCG measure in the context of GS using phenotype predictions evaluated under a simple cross-validation scheme, which is commonly used for benchmarking GS methods [12, 14, 23]. More complex datasets, for example those spanning multiple years or environments, are often assessed using leave-one-year-out or leave-one-environment-out cross-validation, and these schemes are typically evaluated using Pearson’s correlation coefficient *r* [59–63]. In such scenarios, NCG could likewise be applied as an evaluation measure by calculating it within each fold and, if appropriate, averaging across the folds. Importantly, the NCG measure requires only a list of true and predicted values that can be ranked within a selection problem. Its application is therefore not restricted to phenotype prediction and could equally be applied to the prediction of breeding values or other non-breeding-related data. Furthermore, it does not require any assumptions about the underlying distribution of the predicted values to yield an interpretable result, unlike *r*, which requires the normal distribution of breeding values for genetic gain prediction. More broadly, the related NDCG measure originated in the field of information retrieval [24] and has since been employed across diverse domains, including the evaluation of the accuracy of seizure spread prediction [64] and the performance of recommender systems in social media [65].

## Conclusions

In this study, we introduced the normalized cumulative gain (NCG) as an evaluation measure for genomic selection (GS) models. Compared to the commonly used Pearson’s correlation coefficient (*r*), NCG offers several important advantages. While *r* and similar measures assess the association between predicted and true phenotypes, they do not directly address the primary objective of GS: selecting individuals with the highest phenotypic values. In contrast, NCG explicitly measures selection effectiveness by evaluating only the individuals that would actually be chosen. This provides breeders with a more intuitive and easily interpretable metric of selection efficiency. Moreover, we demonstrated that evaluating NCG across all possible selection thresholds allows for a more nuanced comparison of the model performance, revealing differences in model performance under different selection intensities that would be missed by metrics that ignore the selection process, such as *r*, or by calculating NCG for only a few selected thresholds. The NCG measure can provide a valuable advantage when implemented in practical breeding pipelines, as it offers a more interpretable assessment of the performance of a prediction model for a given selection threshold. As the success of a breeding program critically depends on the chosen selection threshold, NCG can further support the decision on an optimal selection threshold. Overall, incorporating NCG into the evaluation of GS models enhances interpretability and offers richer insights into the performance of GS models. Our approach is implemented in R and the script as well as the datasets are available at https://github.com/FelixHeinrich/GS_Comparison_with_NCG.

## Data Availability

The implementation of NCG and the datasets used in this study are available from https://github.com/FelixHeinrich/GS_Comparison_with_NCG.
